# Emerging Perspectives on Gene Therapy Delivery for Neurodegenerative and Neuromuscular Disorders

**DOI:** 10.3390/jpm12121979

**Published:** 2022-11-30

**Authors:** Cintia Gomez Limia, Megan Baird, Maura Schwartz, Smita Saxena, Kathrin Meyer, Nicolas Wein

**Affiliations:** 1 Center for Gene Therapy, Abigail Wexner Research Institute at Nationwide Children’s Hospital, Columbus, OH 43205, USA; 2 Biomedical Sciences Graduate Program, The Ohio State University College of Medicine, Columbus, OH 43205, USA; 3 Department of Neurology, Inselspital, 3010 Bern, Switzerland; 4 Department of Pediatrics, The Ohio State University, Columbus, OH 43205, USA

**Keywords:** neuromuscular diseases, neurodegenerative diseases, molecular biology, CSF delivery, therapeutic strategies, gene therapies

## Abstract

Neurodegenerative disorders (NDDs), such as Alzheimer’s disease (AD) and Parkinson’s Disease (PD), are a group of heterogeneous diseases that mainly affect central nervous system (CNS) functions. A subset of NDDs exhibit CNS dysfunction and muscle degeneration, as observed in Gangliosidosis 1 (GM1) and late stages of PD. Neuromuscular disorders (NMDs) are a group of diseases in which patients show primary progressive muscle weaknesses, including Duchenne Muscular Dystrophy (DMD), Pompe disease, and Spinal Muscular Atrophy (SMA). NDDs and NMDs typically have a genetic component, which affects the physiological functioning of critical cellular processes, leading to pathogenesis. Currently, there is no cure or efficient treatment for most of these diseases. More than 200 clinical trials have been completed or are currently underway in order to establish safety, tolerability, and efficacy of promising gene therapy approaches. Thus, gene therapy-based therapeutics, including viral or non-viral delivery, are very appealing for the treatment of NDDs and NMDs. In particular, adeno-associated viral vectors (AAV) are an attractive option for gene therapy for NDDs and NMDs. However, limitations have been identified after systemic delivery, including the suboptimal capacity of these therapies to traverse the blood–brain barrier (BBB), degradation of the particles during the delivery, high reactivity of the patient’s immune system during the treatment, and the potential need for redosing. To circumvent these limitations, several preclinical and clinical studies have suggested intrathecal (IT) delivery to target the CNS and peripheral organs via cerebrospinal fluid (CSF). CSF administration can vastly improve the delivery of small molecules and drugs to the brain and spinal cord as compared to systemic delivery. Here, we review AAV biology and vector design elements, different therapeutic routes of administration, and highlight CSF delivery as an attractive route of administration. We discuss the different aspects of neuromuscular and neurodegenerative diseases, such as pathogenesis, the landscape of mutations, and the biological processes associated with the disease. We also describe the hallmarks of NDDs and NMDs as well as discuss current therapeutic approaches and clinical progress in viral and non-viral gene therapy and enzyme replacement strategies for those diseases.

## 1. Introduction

Neurodegenerative diseases (NDDs) are a group of heterogeneous disorders affecting the central nervous system (CNS) characterized by neuronal death. Cognitive and motor functions are affected, causing widespread socio-economic burden. A subset of NDDs exhibit both CNS dysfunction and muscle degeneration, as observed in Gangliosidosis 1 (GM1) and Parkinson’s disease (PD), among others. Additionally, NDDs can be associated with age-related dysfunction in the CNS, as seen in Alzheimer’s disease (AD), amyotrophic lateral sclerosis (ALS), Huntington’s disease (HD), and Parkinson’s disease (PD) [1]. In recent years, the global burden of deaths due to neurological diseases has increased approximately by 40%, and disability-adjusted life-years (DALYs) have increased by around 15%. In some conditions, the identification of the causes of these diseases remains challenging [2,3].

Neuromuscular disorders (NMDs) are characterized by progressive muscle weakness in addition to CNS dysfunction. Peripheral nervous system (PNS) and neuromuscular junction (NMJ) degeneration led to muscle weakness in these patients. Amongst NMDs, some disorders are fatal such as Duchenne Muscular Dystrophy (DMD), Spinal Muscular Atrophy (SMA), and Pompe disease. The overall prevalence of NMDs is less than 40 in 200,000 individuals, attributing the conditions as rare [4].

Most NMDs, and some NDDs, are genetically inherited as recessive or dominant mutations [5]. Understanding of the mutational landscape in these individuals has increased with the implementation of new genomic sequencing technologies such as Next Generation Sequencing (NGS), Whole Exome Sequencing (WES), and others. Mutations are often sporadic or occur de novo, both of which can lead to the development of the disease and the symptoms observed in the patients [4,6,7,8]. Genetic mutations can impair the specific function of proteins or enzymes necessary in critical biological processes associated with the pathogenesis of these diseases.

Over the past few decades, several therapeutic approaches for the treatment of NDDs and NMDs have been tested in preclinical studies and have progressed to clinical trials. Tough promising results have been achieved, some limitations of current treatments have been identified. Several studies note the intrinsic low capacity of small molecules or drugs to traverse the physiological barrier, the blood–brain barrier (BBB), as a critical problem. Induction of the immune response during treatment, the persistence of treatment, and the toxicity in peripheral organs, such as the liver and kidneys, affect the efficiency and safety of treatments [9,10,11].

Several therapeutic strategies aim to deliver small molecules or drugs to target specific genes or proteins. Therapeutic strategies currently under evaluation include stem cell therapy, enzyme replacement therapy (ERT), RNA or DNA-based therapy (antisense oligonucleotides (AONs)), and viral vector-based gene replacement therapy including Adeno-associated viruses (AAVs) [12,13,14]. To transport AONs for non-viral gene therapy, systems such as cationic polymers and cationic lipids can be used to generate nanoparticles which can help improve stability, transport, and uptake.

For over 20 years, viral vectors have been used in gene replacement therapy for human diseases, including NDDs and NMDs. For widespread targeting of tissues, the current state-of-the-art viral vectors are AAVs due to their ability to efficiently spread in the nervous system and muscle, their advantageous safety profile over other viruses as well as their non-integrative nature, which reduces the risk of genotoxicity [14]. Multiple AAV-mediated gene therapy clinical trials for NDDs and NMDs are currently ongoing (Table 1) [15]. AAVs can be administered via systemic or cerebrospinal fluid (CSF) delivery in order to target multiple organs and tissues for gene replacement or knockdown of toxic mutated proteins, depending on the specific disease mechanism being addressed. Several studies have identified the main characteristics of different AAV serotypes, their biodistribution, and cellular tropism. Numerous preclinical studies in mice and large animals have evaluated the safety, minimum dose effectiveness, and tolerability of these treatments.

Potential routes of administration for gene therapies include direct CSF delivery via intracerebroventricular (ICV), intrathecal (IT) (including intra-cisterna magna (ICM) and lumbar puncture), and intraparenchymal (IP) administration [16]. Although some CSF delivery routes require invasive procedures, an increase in clinical benefits has been observed due to a significant improvement in the targeting of cells of the CNS as compared to systemic intravenous delivery [17]. CSF delivery may assist in the evasion of pre-existing neutralizing antibodies, a limitation identified in current viral vector-based treatments after systemic delivery. Several preclinical and clinical studies aim to use minimally invasive IT administration for the treatment of neurodegenerative disorders [18]. Initial studies have shown a delay in the progression of disease in patients treated via IT delivery [19]. The biodistribution in the peripheral organs and tissues and tropism for distinct subsets of cells or structures in the brain can also substantially improve after CSF delivery [16,20].

In this review, we discuss important basic AAV biology and vector design elements, therapeutic delivery routes and their advantages and disadvantages, and clinical progress in viral and non-viral gene therapy and enzyme replacement strategies for NDDs and NMDs. We focus on the advantages of CSF delivery and the benefits/limitations of current AAV-mediated gene therapies in clinical trials. AAV-mediated gene therapy has proven to be a promising approach for diseases that have no efficient treatments to date.

## 2. AAV-Mediated Gene Therapy—Considerations for Designing a Therapeutic Approach

AAVs are a tool in gene replacement therapy. With slight modifications to the native virus, they are non-pathogenic and generally safe for humans. These recombinant vectors can efficiently target different tissues due to the availability of naturally occurring and newly engineered capsid proteins, or serotypes, each with their unique affinities for certain cell receptors. Selection of an appropriate serotype must also be complemented by the route of administration, as not all serotypes are able to cross the BBB. Finally, the efficiency of transgene expression within host cells after the vector is internalized is heavily influenced by the structure of the vector genome, the type of cellular promoter utilized, and other regulatory elements. Below we describe in detail these components of recombinant AAVs with emphasis on current and emerging characteristics of vectors utilized in the NDD and NMD field [21].

Currently, many factors are considered in the experimental design of preclinical and clinical trials, including serotype, route of administration, tropism, CNS biodistribution, and the class of the AAV genome. Several studies have evaluated the efficiency of transduction and transgene expression by comparing single-stranded AAV (ssAAV) or double-stranded self-complementary AAV (scAAV) side-by-side in different types of cells/tissues, such as liver, muscle, and brain, among others. AAV can transduce specific cells to different extent, from twenty-five to several hundred vector genome-containing particles (VG) [22].

### 2.1. The Basics of Native AAV Vector Biology

**Figure 1:**
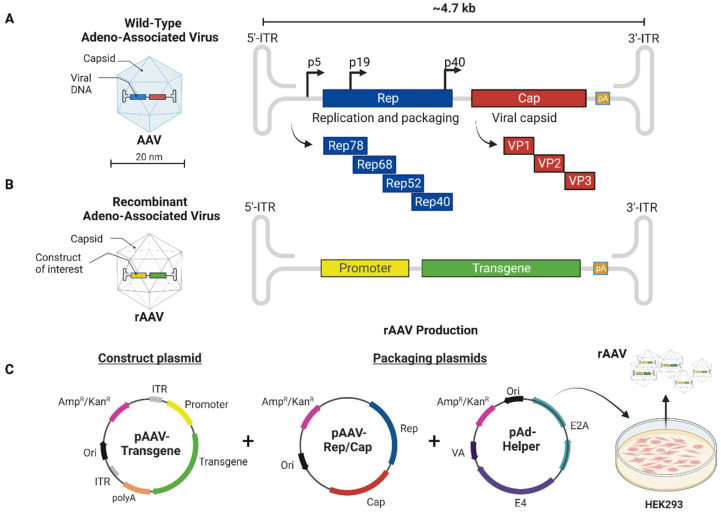
Recombinant AAVs are used for gene therapy. (**A**) The wild-type AAV consists of an icosahedral capsid and a viral DNA genome which includes the viral rep and cap genes. Rep gene encodes the proteins Rep78, 68, 52, and 40 under the promoter p5 or p19. Cap genes encode the proteins VP1, VP2, and VP3 under the promoter p40; both viral genes are flanked between two palindromic inverted-terminal repeats (ITRs). (**B**) Recombinant AAV (rAAV) consists of a capsid that contains the construct of interest. The construct plasmid encodes the transgene under a specific promotor and is flanked between two ITRs. (**C**) rAAV production is performed by co-transfection of two packaging plasmids and the construct plasmid (pAAV-Transgene) using host cells (e.g., HEK293). One packing plasmid encodes the rep and cap genes of a specific AAV serotype (pAAV-Rep/Cap) and the second plasmid is called helper (pAd-Helper), which expresses adenovirus helper genes (E2a, E4, VA). The transgene packaging capacity of a ssAAV is 4.4–4.7 kb.

AAVs are non-enveloped viruses belonging to the Parvovirus family, containing a single-stranded DNA genome (ssDNA) of 4.7 kb and an icosahedral capsid. The replication of wild-type AAVs depends on a co-infection with a helper virus such as adenovirus (AdV), herpes simplex virus (HSV), or Human Papilloma Virus (HPV). In the absence of a helper virus, AAV is unable to initiate productive infection. The viral DNA genome includes two open-reading frames (ORFs), the first encoding four Rep proteins with a central role in replication. The second ORF encodes three subunit capsid proteins (VP1, VP2, and VP3) encoded by the cap gene. Finally, the viral DNA genome is flanked on both sides by two palindromic inverted-terminal repeats (ITRs) that each contain 145 bp T-shape secondary structures and play a central role in the replication process, second-strand synthesis, packaging, and virus production (Figure 1A).

Internalization of AAV into a host cell begins with the molecular interaction between the capsid and cell surface receptors. Additional interaction with a co-receptor may be necessary, depending on the type of cell. After being internalized via clathrin-mediated endocytosis into the cytoplasm of the host cell and taken up by endosomes, the AAV particles can have one of two fates after this point [23,24,25,26,27,28,29]. In the first scenario, the capsid and the genome of the AAV are degraded by proteolysis in the proteasome. The AAV fragments are then presented by the major histocompatibility complex (MHC) class I molecules to immune cells. In this case, antibodies against the AAV capsid and transgene can be generated by the adaptive immune response, which might impact the efficacy of gene therapy. Therefore, the proteasome-mediated vector degradation impedes the transcription of AAV in the nucleus. In the second scenario, after the AAV escapes from the endosome, it can translocate into the nucleus via the nuclear pore complex. At this point, the capsid disassembles and the ssAAV genome is guided by the ITR sequences to synthesize the complementary second strand of DNA by using the host DNA polymerase [27,28,29,30,31].

### 2.2. The Basics of Recombinant AAV Vector Biology

To further enhance the safety profile of AAVs for use in humans, the vector genome was modified to prevent replication and integration into the host genome by removing the *rep* and *cap* genes from the AAV genome. This also allows for the insertion of specific transgene DNA resulting in a recombinant AAV (rAAV). The ITRs, however, are an important component that remains within the genome (Figure 1B). The ITR sequences have a crucial role in synthesizing the rAAV genome and help to circularize the rAAV genome. This episomal structure is able to persist in the nucleus to stably express the transgene in the cell, resulting in long-term expression in dividing and non-mitotic cells without integration into the host genome [23,24,25].

Due to absence of the rep and cap genes, these viral vector constructs do not produce any new infectious virus particles once they reach the target cells. For production, the rAAV replication and encapsulation in the host cells (e.g., HEK293, HeLa) is dependent on providing rep and cap proteins *in trans* for replication. Although several production systems have been developed, the traditional production depends on a co-transfection of several plasmids in HEK293 cells [32]. The first plasmid (pAAV) contains the transgene flanked by ITRs sequences (*cis*-element). In most cases, the ITRs are derived from AAV2. A second plasmid encodes rep and cap proteins (*trans*-elements). The cap proteins will determine the cell and tissue specificity as they build the capsid structure of the virus. The third plasmid (pAAV helper) carries the genes encoding critical proteins that allow the virus to propagate [33] (Figure 1C).

### 2.3. Advantages and Disadvantages of Self-Complementary rAAV Genomes

rAAVs can be designed to include either a ssAAV or a double-stranded, self-complementary genome (scAAV) [34]. Each has its advantages and limitations in terms of the level and onset of transgene expression and transgene packaging capacity. ssAAV will package either sense (plus-stranded) or antisense (minus-stranded) single-stranded genomes, where either strand can use the DNA machinery of the host cell to convert the single-stranded genome into double-stranded for initiation of transcription [35]. This step is considered a rate-limiting factor because transgene expression is directly dependent on the success of single-stranded conversion. The transgene packaging capacity of a ssAAV is 4.4–4.7 kb [36,37].

To improve the rate-limiting step of the second-strand synthesis, scAAV vectors were designed. In brief, the self-complementary genome contains a single-stranded DNA genome in which one of the two ITRs is mutated and the sequence of one-half of the vector is complementary to the other half. Thus, due to the sequences being complementary, the DNA folds to itself and builds a double-stranded structure, able to be used for transcription immediately; however, this reduces the size of the transgene that can be delivered (~2.3 kb) [34,37,38].

### 2.4. AAV Serotype Characteristics and Tropism

The capsid of AAV displays an affinity for certain cells or tissues, which defines tissue tropism, neurotropism, capacity to bypass the BBB, and antigenic properties. Multiple AAV serotypes can transduce specific cells, such as neurons and astrocytes, hepatocytes, cardiomyocytes, muscle cells, and additional organs. Currently, more than 12 different serotypes of AAVs, such as AAV1-9 and AAV10rh have been isolated in humans and other species (Table 2). In addition, the field of capsid evolution has exploded in the last years, and it is likely that hundreds if not thousands of new capsids will become available for future applications [39].

AAV2 was the first serotype identified as a replication-defective vector in 1966, and its genome was sequenced around 15 years later. AAV tropism and CNS distribution in mice, large animals, and humans have been extensively studied. AAV2 shows efficient transduction and long-term expression in dividing and non-dividing muscle cells after systemic or intramuscular (IM) injections in large animals. However, the pre-existing neutralizing antibody in the population impacts the efficiency of the AAV2 for gene therapy. Moreover, AAV2 cannot cross the BBB efficiently after intravenous (IV) administration. AAV2 can be transported inefficiently through endothelial cells in the BBB by passive diffusion [43]. Comparative studies between AAV2 and AAV9 using a reporter transgene showed that AAV2 is also less efficient in muscle transduction after systemic administration [44]. AAV2 is used in clinical trials to treat several NMDs and NDDs patients with local delivery methods. However, AAV9 and close relatives are currently the gold standard in clinical trials with systemic delivery approaches [23,45], including the systemic delivery to the heart [46,47,48,49,50,51,52,53,54]. Due to the ability to cross the BBB after systemic administration, the high efficiency of transduction in muscle cells (skeletal muscles, cardiomyocytes) and neuronal/non-neuronal cells, AAV9 is a highly attractive and popular serotype for clinical trials [55,56,57]. AAV9 can be delivered systemically IV or via CSF injection.

Some AAVs migrate by using the same axonal trafficking pathways, which are used to transport organelles, lysosomes, and plasma membranes. rAAV is transported by synaptic vesicles in the neurons through the microtubule network (MT). These MTs have polar ends, a plus (+) end terminal (dimerization of alpha beta-tubulin) or minus (−) end terminal (depolarization of tubulin dimer). Retrograde transport of rAAV begins at the axon terminal and traffics toward the soma of the neuronal cell body. By the utilization of dynein motor protein, transportation is directed to the minus (−) end terminal [58]. AAVs capable of retrograde transport include AAV1, AAV2, AAV5, AAV6, AAV7, AAV8, AA9, and AAVrh10. In contrast, anterograde axonal transport traffics the rAAV from the soma to the axon terminal. By utilization of kinesin motor protein, transportation is directed to the plus (+) end terminal. AAVs capable of anterograde transport include AAV1, AAV2, AAV5, AAV7 and AAV9. AAV9 is unique, as this serotype is capable of bidirectional transport [59]. In types of axonal transport, AAVs can migrate short or long distances from the area of injection around the CNS and PNS. These characteristics contribute to the understanding of the CNS distribution of rAAVs and associated neurotropism [56].

### 2.5. Cell-Specific and Ubiquitous Promoters—Modulating Gene Expression

Different types of promoters and enhancers have been selected in combination with regulatory elements to drive the transgene expression. The promoter enables the preferential expression of the transgene in a specific cell type or tissue; therefore, the selection of the promoter is based on the type of cells and tissues necessary to target. Ubiquitous promoters can express the transgene in various organs and tissues simultaneously. Several viral promoters are currently in use including cytomegalovirus (CMV) and Rous sarcoma virus (RSV). CMV has shown a constitutive and high expression in muscle cells. It is important to verify the long-term expression of promoters as especially viral promoters are at risk of being shut down by the cellular immune response, which can reduce the long-term efficacy of treatment. There are also eukaryotic constitutive promoters able to express transgenes in a wide variety of cell types. This class of promoters includes elongation factor 1 alpha (E1alfa), phosphoglycerate kinase (PKG), ubiquitin C (UBC) and chicken beta-actin promoter. The latter is often combined with a CMV enhancer (CAG). Other promoters express the transgene in specific cells or tissues of interest; for example, for the nervous system for expression in neurons, PDGF-β promoter, human synapsin 1 (hSyn1) promoter, and α-CaM Kinase II are frequently used [60,61,62].

Promoters that exclusively express the transgene in muscle cells include muscle creatine kinase (MCK), desmin (Des), or alpha-myosin heavy chain (a-MCH). A new variation of α-MCH was designed in order to improve muscle transduction—MHCK7 (770 bp), which is formed by a hybrid of MCK and alpha-MHC promoter. The specificity of this promoter has been shown in both skeletal and cardiac muscles [63].

### 2.6. Engineered AAVs with Enhanced Tropism to Muscles and CNS

In recent years, several groups have engineered new AAV serotypes to improve the efficiency of tropisms in the whole body and CNS. Neurotropism has been explored in mice and large animals in the brain and spinal cord. The engineered capsid of AAV is generated by modifying the parental capsid in order to improve the transduction efficiency in specific cells.

The technologies to modify the capsids include different approaches, such as the Cre recombination-based AAV-targeted evolution (CREATE) technique. Wild-type (WT) capsids can also be altered by chemical modification, peptide insertion, and capsid shuffling, among others. In the following, we point out a few examples that show promise for muscle and nervous system delivery.

Engineered serotypes such as AAV.PHP.B and AAV-B1 have shown a higher efficiency in crossing the BBB and transducing neuronal cells. AAV.PHP.B can bypass the BBB 40 times more efficiently than AAV9 and transduce neurons, astrocytes, oligodendrocytes, and non-neuronal cells in mice. However, AAV.PHP.B did not show the same advantages in large animals that lack the L6y surface receptor.

Another group of new AAV-engineered capsids has shown an efficient delivery in the heart [64,65].

In order to improve the tropism in the CNS, other engineered capsids were generated, such as AAV2/AAV8 chimera capsid, which is called AAV2i8. AAV2i8 can target selectively and efficiently the cardiac and skeletal muscles. In addition, AAV2i8 presents a low diffusion to the liver compared to the parental strains. This AAV is a promising serotype for gene therapy to the heart [66]. Another novel capsid derived from AAV9 is termed AAV9.45. This serotype shows 10 to 25-fold lower diffusion in the liver than the parental strain. Thus, this AAV capsid is a potential candidate that might reduce the safety issues observed at high IV doses [67]. AAV-B also has been shown to improve CNS delivery; however, this capsid has also exhibited substantial tropism for muscles as well as peripheral organs such as the pancreas and lungs. Notably, there is a reduced immune response of neutralizing antibodies in pooled human serum for this novel serotype [68].

AAV-AS is also able to transduce neural cells in the CNS after systemic delivery. AAV-AS improved the neural transduction around 6 to 15-fold compared with its parental AAV9 and its efficacy was recently evaluated in a Huntington mouse model [69].

AAVM41 was derived from AAV1, 6, 7, and 8 and has shown tropism for the heart and muscles and presented a low delivery in the liver. This AAV is a candidate for cardiac muscle cells [70]. AAV2.retro and AAV2-HBKO, showed an efficient retrograde transport in mice and large animals [71,72].

In order to increase the tropism and transduction efficiency of muscle fibers, AAVpo1 was generated [73]. This serotype was isolated from pigs suggesting a better application in gene therapy in terms of the immune response. A study demonstrated that no neutralizing antibodies were identified against AAVpo1 capsid, even in blood containing antibodies against serotypes 2–8 [73]. Later, new variants such as AAVpo2.1, a hybrid capsid of AAVpo2 and AAVpo6 were selected in order to obtain a capsid with lower transduction affinity for hepatocytes and high transduction efficiency in muscles, kidney, and spleen [74].

### 2.7. AAV Delivery and Immune Response

High seroprevalence of natural AAV has been identified in around 80–90% of the human population. Multiple serotypes have been isolated from humans, NHP, and pigs, among other species. Around 40–60% of the population are positive for pre-existing anti-AAV antibodies against serotypes 1, 2, 3, or 5 [75]. These pre-existing anti-AAV antibodies can neutralize several AAV serotypes. The cross-reactivity among different serotypes can negatively influence the delivery and cell transduction of AAV used for therapy and can cause severe immune reactions. Different strategies to evade immune responses have been established in clinical settings to avoid a strong immune response after AAV administration, including different cocktails of anti-inflammatory drugs given prior to or after the dosing of the patients [76,77].

Entry of peripheral immune cells to the CNS is impeded by the BBB. The possibility of using CSF delivery to evade the peripheral immune response has been suggested; however, this suggestion is still controversial due to studies demonstrating that transgene expression in the CNS is still inhibited by pre-existing neutralizing antibodies [78]. On the other hand, other studies confirm the possibility of evading the neutralizing antibodies using CSF delivery [9].

## 3. Routes of Administration

### 3.1. Local Administration

For both NDDs and NMDs, local administration has and continues to be explored for both AONs and AAV viral vectors. IM serves as a convenient and non-invasive route to target the PNS or for targeting of muscle. However, the efficiency of this route of administration in skeletal muscles is limited as lower doses need to be used due to limitations in injection volumes and to avoid local toxicity at the injection sites [79]. Importantly, even when AAVs are injected into a certain muscle, they still tend to spread into the blood stream. There might also be some transport to the CNS depending on the AAV capsids used. Unfortunately, the spread is not sufficient to generate a therapeutic effect due to the overall lower doses used. Although AONs are a good alternative to AAVs, they are not very efficient in spreading to other muscles or the CNS when delivered IM. For CNS diseases, AONs or AAVs can be delivered by intraparenchymal (IP) injections directly to specific affected brain regions such as the thalamus, for example. While IP injections are well suited for the treatment of isolated brain regions, they are not effective when widespread targeting is needed due to limitations in injection volumes that limit the overall doses that can be delivered. Moreover, local inflammation and toxicity have been reported in several clinical trials that could be related to either the injection procedure or to the locally high vector concentration. Another complication is the accessibility of the brain and the need to insert needles through the skull and all the way through the brain to the location of interest. Such procedures are complicated and represent a significant risk.

### 3.2. Systemic IV Delivery vs. CSF Delivery

Due to the limitations of local administration described above, many preclinical and clinical trials use systemic administration as the main route of delivery to investigate potential treatments for NDDs and NMDs. This type of administration includes IV administration.

IV administration of drugs or small molecules allows widespread targeting of different muscle groups throughout the body as the treatments are distributed thanks to the blood stream. In order to obtain a therapeutic effect, the doses used are usually substantially higher compared to IM injections, given that multiple muscle groups need to be targeted and dosing is calculated per kilogram body weight. However, as the blood carries the therapeutics, extensive spread to other internal organs is also seen, in particular the liver and kidneys. This unfortunately increases the risk of toxicity in peripheral organs, such as the liver and kidneys, and may also trigger the host immune response and cause adverse safety events.

Thus, while IV treatment is suitable for infants and younger children, AAV therapies become more challenging for the treatment of teenagers or adults due to high vector production costs as well as potential safety concerns at very high doses. Since IV delivery results in high targeting levels of the liver, severe adverse events have been observed in older patients at high doses related to liver failure, heart inflammation, and kidney. In the last few years, several patients in clinical trials for Duchenne Muscular Dystrophy and Myotubular Myopathy have passed due to these side effects.

Many neuromuscular disorders also display pathology of the nervous system (NS) and several AAV serotypes are able to cross the BBB. While IV delivery of AAV has shown promise to target the NS, for example, for Spinal Muscular Atrophy (Zolgensma), the efficiency is still low for serotypes currently used in clinic, requiring high doses; therefore, efficient simultaneous targeting of both the muscle and NS via CSF would likely enhance therapeutic outcomes.

CSF flows within cerebral ventricles, cisternal spaces, the subarachnoid space and is a preferred route of delivery for gene therapy to brain and spinal cord [80]. Importantly, CSF delivery overcomes the challenges mediated by the BBB. Moreover, direct CSF delivery of gene therapy also attenuates the potential risk of circulating neutralizing antibodies as well as vector-associated toxicity observed during systemic delivery of high concentration of viral vectors [81].

Several studies have evaluated the efficiency of delivering gene therapy vectors through CSF instead of IV injections for NMDs to target both muscle and the NS [82,83]. Importantly, CSF delivery allows the targeting of both the NS as well as muscles and peripheral organs with AAV. A significant amount of AAV vector will leak into the periphery and targeting of the liver and other organs is still high; however, thanks to better access to the CNS, the CSF-delivered doses are magnitudes lower compared to IV doses, which as a consequence, also automatically reduces the amount of virus that makes it to the liver. Moreover, when using CSF delivery, the dose is generally not adjusted to the body weight of the patient as the brain and spinal cord do not show the same weight and size difference between patients compared to muscle mass. Hence, researchers use other measures such as CSF volume, brain weight, or the number of neurons to estimate and escalate dosing from preclinical models to human patients.

**Figure 2:**
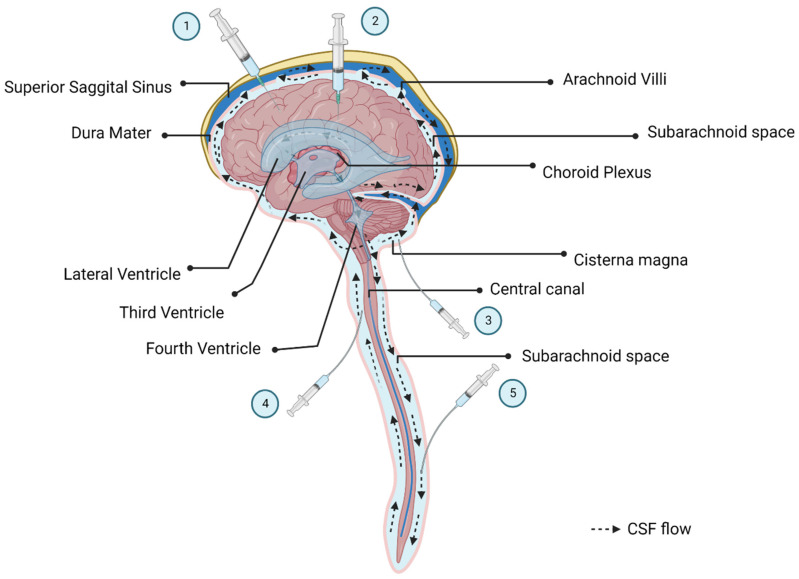
Normal human CSF circulation and types of CSF injections. CSF is secreted by the choroid plexus into the lateral, 3rd, and 4th ventricles. CSF then flows to the subarachnoid space in two directions. The first is towards the intracranial subarachnoid space surrounding the brain. The second is towards the spinal subarachnoid space in the spinal cord. After the CSF completes circulation in the spinal cord, it then flows back to the brain. CSF drains through arachnoid villi to the venous sinuses (superior sagittal sinus) and into venous blood. Black arrows indicate the normal circulation of CSF in humans The injection sites along the CNS are indicated from numbers 1 to 5. 1—Intrapharenchymal (IP) administration, 2—Intracerebroventricular (ICV) administration, 3—Intracisterna magna (ICM), 4—Intrathecal (IT) injection in the cervical region of the spinal cord, 5—Intrathecal (IT) injection in the lumbar region of the spinal cord.

As mentioned above, there are several access routes to the CSF that are used in preclinical models and in clinical trials (Figure 2). ICV access requires passing a needle through the skull to access the ventricles. This is a relatively invasive procedure and is complicated, especially for repeat dosing such as ERT or AON delivery; however, it is possible to install a port system that can be more easily accessed. Studies have shown that a lower dose is necessary for ICV delivery compared to IV injection to achieve widespread targeting of the brain. Though ICV injection has been shown to be safe for use in humans, the high invasiveness of this procedure should be considered [84]. The rate of infusion and volume limitations need to be controlled as cranial pressure can increase after injections. There is also a risk of parenchymal hemorrhage after ICV injection [84].

IT injections deliver the therapeutics agent into the spinal canal or the subarachnoid space. Several injection sites can be leveraged to gain access to the CSF via that route. One frequently used method is the cisterna magna (*CM; also known as a posterior cerebellomedullary cistern*) puncture. The CM is located between the cerebellum and the medulla oblongata. The access does not require penetrating the skull; however, ICM injections are invasive and procedural risks include injury to vascular structures and the brainstem [85,86].

As an alternative to the CM injection route, IT injections can also be performed at different positions in the spinal cord (cervical, thoracic, or lumbar) (Figure 2). IT injections in the lumbar spinal cord area are routinely performed in mice, large animals such as NHPs, and humans. To improve the diffusion of the virus to the brain after lumbar puncture, subjects can also be placed in the Trendelenburg position with their head tilted downwards [87]. Preclinical studies have shown that tilting the subject 15–30% significantly increases AAV transduction of the brain as well as the cervical and thoracic spinal cord [83]. Lumbar IT (LIT) injections are a widespread routine injection route, which makes these approaches attractive for clinical trials. In order to further spread the virus towards the brain, some clinical trials use catheters that are inserted in the lumbar spinal cord and threaded up to the thoracic or cervical spinal cord area.

While overall, CM and IT delivery of AONs, ERT, and AAVs seem to be well tolerated in animals and human patients, several risks and/or adverse events were described. For example, the use of catheters at the time of injection can lead to tissue damage or hemorrhages. LIT injection presents a lower risk compared with the other type of injections; however, epidural hematoma can occur (after single or multiple administrations). In addition, CSF delivery at any access point can cause CSF leakage, which can cause headaches, nausea, neck stiffness, and light sensitivity. Other adverse events include bleeding, and pain [86,88,89].

The efficacy of each of the above-described delivery methods, determination of minimally effective dose, long-term expression, and safety/tolerance have been evaluated previously in many preclinical AAV studies. In addition, the biodistribution of AAV serotypes via each delivery route has been characterized by multiple groups [18,28,90,91]. However, a direct comparison between different studies is complicated as details such as injection volume, viral vector formulation, injection speed, and anesthetics used vary and are not always described in detail.

### 3.3. Disruption of BBB to Improve Systemic Delivery

To increase the efficiency of targeting the cells of the CNS via the IV route, mannitol has been used to disrupt the BBB and allow for greater AAV transduction [92].

**Figure 3:**
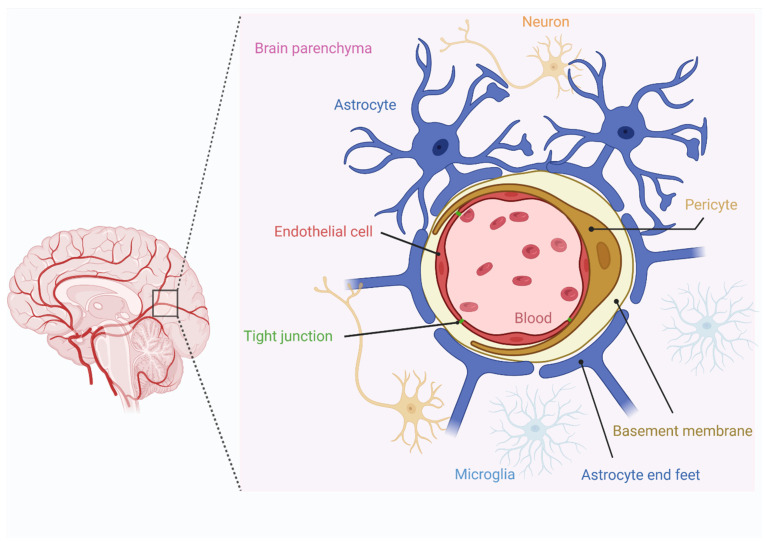
Overview of the blood–brain barrier (BBB). The BBB is a critical component of the neurovascular unit (NVU), which contains neurons and interneurons, microglia, astrocytes (end feet), endothelial cells, and pericytes found around the brain capillaries. Microglial cells protect the brain from microorganisms by phagocytosis. Pericytes surrounded endothelial cells and contributed to the integrity of the BBB. A basement membrane is found between the astrocyte (end feet) and the pericytes. Endothelial cells (ECs), connected by tight junctions (TJs), also contain transporters and receptors. TJs regulate the transport of molecules and ions to the brain. ECs transport nutrients and regulatory molecules (influx/eflux) to the brain through passive or active mechanisms. Cell–cell interaction can regulate numerous functions in the brain, including those important for cerebral homeostasis.

The BBB is a physical barrier surrounding the CNS, consisting of complex tight junctions between adjacent endothelial cells, providing protection from serum factors and other circulating toxins (Figure 3). Mannitol is used for both small molecules and AAV treatments to improve the delivery of drugs to the CNS. Mannitol disrupts the BBB via osmotic pressure in the endothelial cells after systemic delivery as an intra-arterial injection [93]. As an alternative to mannitol, ultrasound (US) is used as a method to induce a temporal disruption of the BBB. By sonication (single or multiple applications), this method generates acoustic pressure and forms gas microbubbles to disrupt the BBB [94]. Several preclinical studies have been performed to evaluate the safety and translational capacity of BBB disruption. The side effects observed are extravasation of red blood cells and petechial bleeding, and inflammatory response. After US treatment in preclinical studies, AAV1 and AAV2 showed increased efficiency in bypassing the BBB [95]. Several types of equipment were designed to disturb the BBB, such as InSightec, ExAblate 4000 system (currently used in a clinical trial—NCT02343991), CarThere SAS, SonoCloud device (used in a clinical trial—NCT02253212). A traditional method to monitor the BBB disruption is by MRI using gadolinium injection. It is important to take into account the natural BBB breakdown in certain diseases, leading to increased permeability for therapeutic drug delivery in CNS disorders such as multiple sclerosis, AD, PD, and many others [96].

## 4. Selected Neurodegenerative and Neuromuscular Diseases and Clinical Examples for Novel Treatment Approaches Focusing on Gene Therapy

### 4.1. Gangliosidosis 1 Disease

Gangliosidosis 1 (GM1), classified as a lysosomal storage disease (LSD), is a fatal neurodegenerative disorder, affecting individuals of various ages, with the most severe cases occurring in children. The GM1 incidence is 1:100,000 to 1:200,000 live births [97].

GM1 is caused by bi-allelic mutations in the *GLB1* gene resulting in a deficiency of a beta-galactosidase enzyme (β-GAL, EC 3.2.1.23) and is inherited in an autosomal recessive manner. Mutations in the *GLB1* gene result in a partial or complete loss of β-GAL activity [98]. *β-GAL* has a critical role in the lysosomes, participating in the breakdown of glycosphingolipids such as ganglioside 1. *β-GAL* deficiency leads to the accumulation of GM1 ganglioside and the GA1 ganglioside (substrate derived from GM1 ganglioside) in the lysosomes, leading to dysfunctional physiology of neurons and subsequent neuronal death [99]. The accumulation of toxic products activates the immune response and affects mitochondrial function [100]. The level of enzyme activity of β-GAL determines if the phenotype is severe (lower enzyme activity) or mild (higher enzyme activity) in GM1 patients. Gradual motor deterioration is a hallmark of GM1 progression [101].

GM1 patients are classified into three major groups depending on their age and the first symptoms that present in the patients—infantile type 1 (OMIM 230500), juvenile type 2 (OMIM 230600), and adult-onset chronic type 3 (OMIM 230650). This classification system allows for the identification of the severity of disease in these patients. The most severe phenotype of GM1 is infantile type 1, with an onset between birth and up to 6 months of age. A hallmark of infantile type 1 GM1 is seizures, which eventually propagate neuronal degeneration. Peripheral organs, such as the liver and spleen, are also affected. Other symptoms include abnormal facial features and skeletal abnormalities, joint stiffness, and muscle weakness. The infant may become blind and/or deaf before one year of age. Patients with infantile GM1 have an expected survival of up to 2 years [102,103].

The second group is late infantile or juvenile, type 2. Juvenile type 2 GM1 is an intermediary phenotype in which the first symptoms appear around one to five years of age. The progression of the disease is slower compared to type 1. Possible symptoms observed are ataxia, seizures, dementia, and speech difficulty. Patients with juvenile type 2 GM1 have a life expectancy up to mid-childhood or early adulthood. Finally, type 3 GM1 is a chronic, adult form of GM1, defined by symptoms onset generally during childhood or adolescence. The common symptoms of type 3 GM1 are muscle atrophy, corneal dysfunction, and dystonia. Type 3 GM1 exhibits a very slow progression compared to type 1 and type 2 GM1 [97,104].

Currently, there is no Food and Drug Administration (FDA) approved treatment for these patients that could permanently alter the disease course. However, therapies are available to treat some of the symptoms, such as anticonvulsants that are used to regulate seizures in patients. Recently, substrate reduction therapy (SRT) has shown a potential benefit by partially inhibiting the biosynthesis of GM1 gangliosides. This inhibition regulates the rate of biosynthesis and the impaired rate of catabolism. SRT has been utilized in conjunction with N-butyldeoxygalactonojirimycin (NB-DNJ) in patients with juvenile and adult GM1. Results from these studies revealed a delay in the progression of the disease [105].

Bone marrow transplantation (BMT) of allogeneic hematopoietic stem cells (HSCT) has been used as a treatment approach to restore the expression of β-GAL in the lysosomes of a type 2 GM1 patient. This therapy restores the normal function of microglia in the brain via hematopoietic progenitor cells differentiated from a healthy donor. The efficacy of the treatment was only observed for the short term, and the beneficial effects diminished during long-term treatment and debilitating symptoms such as ataxia, or speech and walking difficulties continued to occur [106]. Another existing treatment for these patients is enzyme replacement therapy (ERT). This therapy can help prevent the progression of the disease. When used in conjunction with ERT, physical and occupational therapy provides additional benefits. Due to the inability of β-GAL to cross BBB and target cells of the brain, ERTs are delivered via a plant-made recombinant enzyme fusion with both lectin RTB (ricin toxin B-subunit) and β-GAL activity. The lectin activity works as a receptor signal to mediate the endocytosis of the recombinant protein into GM1 fibroblast cells, and the β-GAL restores the enzyme activity in these deficient cells. This approach showed promising results in vitro [107]. In a recent study using ERT, a recombinant enzyme with β-gal activity was ICV administered in a GM1 mouse model. Mice were evaluated for the biodistribution of the enzyme, which revealed lysosome-located β-GAL recombinant protein in neurons, liver, and bone marrow cells [108].

A Phase I/II clinical trial (NCT03952637) began in 2019 to evaluate the safety and tolerability of rAAV9.GLB treatment. GM type 2 patients between 2 and 12 years of age were administered rAAV9.GLB (AXO-AAV-GM-1) (1.5e+13 vg/kg or 4.5e+13 vg/kg) by IV. In this treatment, immune suppressors were administered before systemic vector delivery, in order to reduce the immune response to the AAV capsid and/or the transgene encoding *β-GAL*. This trial is expected to be completed in 2027 [109,110]. In another clinical trial Phase II/III, which was initiated in 2020, rAAVrh10-GLB1 (LYS-GM101) (8e+12 vg/kg) is delivered via systemic injection in infantile Type 1 GM1 (NCT04273269). This trial is expected to finish in 2030 [111]. A third clinical trial dose-escalation evaluates the safety and tolerability of rAAVhu68-GLB1 (PBGM01) administered via CM injection (Imagine-1, NCT04713475) for the treatment of infantile Type1 and 2 GM1. This clinical trial is expected to finish in 2029. Recently interim safety and biomarkers results were reported for a low dose of rAAVhu68-GLB1 after 13 months or 7 months of treatment for the first patient or second patient, respectively (cohort 1), with no evidence of serious secondary adverse effects (SAE) and no dorsal ganglion toxicity identified, showing a biological effect in these patients [112].

### 4.2. Neuronal Ceroid Lipofuscinosis Type 2 and Other Forms of Batten Disease

Neuronal ceroid lipofuscinosis (NCL) is a group of inherited and autosomal disorders caused by ceroid lipofuscin accumulation in neuronal cells. These disorders affect the brain and retina; patients develop dementia, epilepsy, and loss of vision. There are several forms of NCL. Late-infantile neuronal ceroid lipofuscinosis Type 2 (CLN2-NCL) is one of the NCLs that affects both CNS and motor skills in children [113]. Inherited autosomal recessive mutations (bi-allelic) in the CLN2 gene encode the enzyme tripeptidyl peptidase 1 (TPP1) [114,115]. TPP1 deficiency leads to lysosomal accumulation of ceroid lipofuscin (auto-fluorescent lipopigments) initially affecting the nervous system in infants between two to four years of age [116,117]. CLN2 patients present a progressive loss of vision, cognitive dysfunction such as issues with speech, behavioral problems, seizures, failure in the cardiac system, and movement abnormalities, leading to premature death between 12 to 30 years of age [118].

ERT therapy is the most developed and studied treatment option for CLN2 patients. Several clinical trials have been initiated to study the efficacy of delivering recombinant *TPP1* via an intraventricular access device for injection directly into the CSF [54,119]. In 2017, this treatment received FDA approval under the name Brineura^®^ (BMN190). Several clinical trials and interventional studies have been completed (NCT05152914, NCT01907087, NCT02485899) or are ongoing (NCT05152914) to evaluate the long-term efficacy, safety, or tolerability of BMN190 in patients with CLN2 mutations. A long-term treatment clinical trial injected Brineura® via ICV delivery in children with CLN2. Results showed that the treatment had been well tolerated for up to 5 years. Although a huge step forward, the treatment still has limitations. Patients must receive enzyme infusions every two weeks in order to maintain enzyme levels, since the treatment itself does not instruct cells to make their own enzyme. This is a major limitation that can be resolved by traditional AAV gene replacement strategies, which have also been studied in recent years [120,121].

In 2005, a Phase I clinical trial began enrollment of 11 children to evaluate the safety and efficacy of the gene transfer vector, rAAV2CUhCLN2 (NCT00151216). The therapeutic vector was delivered via direct IP injection (3e+12 vg) via multiple injection sites to access different regions of the brain. Eleven patients were divided into two cohorts: moderate disease stage (disability score of 5 to 6; n = 6) or severe disease stage (disability score 0 to 4; n = 5). The main goal of this clinical trial was to evaluate the persistent expression of TTP1 in the brain in addition to a number of safety outcomes. Final results reported high tolerance to the treatment; however, only a small benefit to the recipients [103,122].

Therefore, the original investigators later initiated another Phase I (NCT01161576) and Phase I/II (NCT01414985) trial utilizing the same transgene but with a different AAV serotype AAVrh10-CNL2 that would allow better diffusion in the tissue. The patients were divided into two groups: low dose (2.85e+11 vg) or high dose (9.0e+11 vg). The injection method remained the same. The study began in 2010 with the aim of evaluating the tolerability and safety of both doses. Initial results using the low dose of the therapeutic vector showed that most patients (n = 9) presented T2 hyperintensities that persisted for 6–12 months post-treatment [123]. These flairs were localized to the vector administration sites and present as persistent edemas. Due to this, the investigators decided to lower the dose to 2.85e+11 vg. Although AAVrh10h-CLN2 therapy slowed progression in some patients, recombinant TPP1 therapy (Brineura^®^), which is the first ERT to be directly administered into the CSF was more efficacious, with a greater reduction in the rate of decline of the same neurologic parameters compared to gene therapy.

Multiple other forms of Batten Disease are currently being explored in AAV gene therapy clinical trials. For CLN3, a Phase I/II study in which patients were administered via LIT with scAAV9.P546.CLN3 (AT-GTX-502) (NCT03770572) is currently underway. Interim results have shown safety and efficacy in three patients [124]. For CLN5, there is a Phase I/II study to investigate the safety and efficacy of the therapeutic vector scAAV9.CLN5 (hCLN5opt) (NGN-101) administered via ICV and IV delivery. A Phase I/II study in which CLN6 Batten Disease patients were injected via LIT delivery using scAAV9.CB. CLN6 (AT-GTX-501) aims to evaluate the safety and efficacy of the therapeutic vector (NCT02725580). For CLN7, two cohorts of patients received sc.AAV.CLN7 via LIT injection with low dose (5e+14 vg) and or high dose (1e+15 vg). This study is a Phase I study that aims to evaluate the safety of the therapeutic vector (NCT04737460). These select Batten diseases have all benefited from gene replacement strategies that are currently being investigated in Phase I or I/II clinical trials. All trials except CLN2 utilize the AAV9 serotype for transgene delivery via lumbar IT injection except for the CLN5 trial, which utilizes intracerebroventricular and intravitreal co-injections. For CLN6 and CLN3, which were the first clinical trials using CSF delivery of AAV for Batten Disease, promising interim data was released by Amicus Therapeutics on several occasions [124].

### 4.3. Parkinson’s Disease

Parkinson’s disease (PD) is a progressive neurodegenerative disorder that primarily affects movement. PD is characterized by the loss of dopaminergic (DA) neurons in the substantia nigra leading to dysfunction of the basal ganglia motor network. PD affects around 1 to 3% of people worldwide over 60 years of age [125]. While several regions of the brain are affected in PD, the substantia nigra is typically the most severely affected. The Substantia nigra pars compacta play a critical role in controlling balance and movement of the body. The hallmark symptoms observed in PD patients are tremors of the hand, arms, legs, jaw, and head. Symptoms are most often observed during slow, controlled movement and often include deficits in balance. Behavioral symptoms are also present, such as emotional instability. In addition, some patients can present with a plethora of other symptoms, including hypotension, sleep problems, and an inexpressive face. As the disease progresses, patients have increasing difficulty walking and speaking, and, in some cases, patients present with dementia [126].

While some patients harbor inherent mutations, other patients present with sporadic mutations across multiple genes. The cause of the disease is still unknown; however, both genetic and environmental factors can influence the development of the disease. Thus, PD is considered a multifactorial disease. There are two main age groups for the onset of symptoms—late onset, with symptoms manifesting around 60 years of age, and juvenile onset, which begins at approximately 20 years of age.

Approximately 10–15% of cases are familial, with both autosomal recessive and autosomal dominant inheritance patterns. Several inherited autosomal dominant mutations have been identified, such as those in gene encoding alpha-synuclein protein (aSYN) (SNCA/PARK1), glucocerebrosidase (GBA), leucine-rich repeat Kinase 2 (LRRK2) (PARK8), PARK3, PARK4, and vacuolar protein sorting-associated protein 35 (VPS35) genes. Autosomal recessive mutations occur in genes such as parkin RBR E3 ubiquitin-protein ligase (PARK2), Parkinson protein 7 (PARK7), and phosphatase and tensin homolog-induced Kinase 1 (PINK1) (PARK6) genes associated with mitochondrial dysfunction. Some of the mutations implicated in familial PD have been identified in patients with sporadic cases. A smaller subgroup of patients presents with mutations in the TAF1 gene, which is associated with X-linked inheritance. In addition, a group of mutations in the genes GBA and UCHL1 (PARK5) are considered a risk factor for PD. Both autosomal recessive mutations and autosomal dominant mutations can cause early onset class juvenile-onset PD [126].

Mutations in *SNCA* (*PARK1*), *LRRK2* (*PARK8*)*, PARK2*, *PARK7,* and *PINK1* (*PARK6*) genes result in misfolding and accumulation of proteins affecting dopamine synthesized in DA neurons. Other mutations can be associated with the generation of ATP in the mitochondria, and the deficiency of these genes can lead to the dysfunction of neurons. Another mutation in these genes can affect the elimination of free radicals in the cells leading to the death of DA neurons [126].

The symptoms of PD manifest due to the selective loss of DA neurons in the substantia nigra, thereby disrupting the connection of substantia nigra neurons that project to the striatum and frontal cortex. Dopamine is a catecholamine neurotransmitter present in the synapses of DA neurons, which under normal circumstances, influences physical movements. In the absence of dopamine, there is dysfunction of the motor circuit, which impacts motor function. Other catecholamine neurotransmitters, such as norepinephrine, are affected in PD, affecting the sympathetic nervous system. The sympathetic nervous system controls autonomic functions in the body, including blood pressure. Because the patients also show symptoms associated with non-motor functions, the lack of norepinephrine can lead to the development of secondary symptoms such as blood pressure failure and fatigue [127]. The disease progression varies widely among patients, with some patients exhibiting severe dysfunction while others may show minor motor deficiency. After treatment with norepinephrine analogs, the patients can show improvement in cognitive functions, hypotension regulations, and sleep issues.

The gold standard of treatment for PD is Levodopa; a dopamine precursor, which was approved by FDA in 1975; however, though it is very effective, it is not a curative treatment [128]. Long-term administration causes tolerance to the treatment. In some cases, patients can present dyskinesia, cognitive dysfunction, and psychiatric issues as side effects of Levodopa administration. An alternative treatment is subthalamic nucleus implantation of electrodes known as deep brain stimulation (DBS), which increases neural activity in specific areas of the brain [129]. However, DBS does not improve speech and cognitive functions in patients [130].

Mutations or polymorphisms in the *SNCA* gene can contribute to the development of PD. Several studies suggest that α-Syn accumulates as amyloid fibrils resulting in Lewy bodies [131,132]. α-Syn also misfolds, thereby aggregating in neurons, leading to neuronal dysfunction [133]. Recently, Cole and colleagues demonstrated AON therapy can reduce the expression of (α-Syn) protein in a rodent PD model, improving DA neuron function in these animals. In addition, the authors showed a wide distribution of the human *SNCA* AONs in the brain of nonhuman primates (such as the cortex, striatum, and midbrain) and identified reduced a-SYN levels in the CSF. Thus, inhibiting α-SYN may be sufficient to ameliorate some of the pathological characteristics associated with PD making *SNCA* AONs a promising disease-modifying therapy for PD patients [134].

Neurons in the putamen are connected to DA neurons in the substantia nigra. The putamen is a potential region of interest for the transplantation of cells. In addition, the putamen is more accessible for injection than the substantia nigra. Thus, the putamen is an attractive target for cell therapy to restore lost DA neurons via either mesenchymal stem or neural stem cells (NSCs) cell engraftment. After implantation of stem cells and the subsequent stimulation with neurotrophic factors in animal models, such as Glial Cell-Derived Neurotrophic Factor (GDNF), the cells are able to regrow in the brain and restore the normal function of DA neurons [135]. However, it is still unknown whether undifferentiated MSCs or neuronal cells derived from MSCs have the ability to incorporate into neural circuits with normal functions [136,137,138].

Several clinical trials have been established to treat PD patients carrying mutations in the SNCA gene; there are three completed studies NCT04273932, NCT03318523, and NCT02459886, and ongoing studies such as NCT05274568, NCT02954978, and NCT05424276.

AAV gene therapy is a promising therapy for PD as AAVs can transduce diffuse regions of the brain, including PD disease-specific brain regions [54]. In PD, neurodegeneration in the substantia nigra leads to dysfunction of dopamine signaling to the striatum. As patients with PD present DA neuron deficiency resulting in impaired motor skills, the stimulation of this specific neural population by GDNF or GAD is a promising therapeutic strategy. There are thirteen clinical trials for PD utilizing AAV2, that contain different therapeutic transgenes such as enzyme Aromatic L-Amino Acid Decarboxylase (AADC), glial cell line-derived neurotrophic factor (GDNF), glutamic acid decarboxylase (GAD), and CERE-120 (neurturin-NRTN) [139].

AADC is the rate-limiting enzyme in the conversion of levodopa to dopamine. To investigate the safety of increasing AADC expression in the striatum, a Phase I clinical trial utilizing AAV2 carrying AADC (VY-AADC01) was initiated in 2013. Patients enrolled in the clinical trial continued to take PD medications, including Levodopa. The treatment is administered via bilateral IP infusion into the striatum. This is a dose-escalation study with a low dose (7.5e+11 vg), intermediary dose (1.5e+12 vg), and high dose (4.7e+12 vg) in a total of fifteen patients. Other phase I/II clinical trials test different doses of VY-AADC01 or VY-AADC02 via injection of either striatum or putamen (NCT03065192, NCT00229736, NCT03733496, RESTORE-1; NCT03562494). RESTORE-1 is a multicenter, Phase II study that was recently completed. The study aimed to evaluate safety and efficacy. Patients were IP injected (striatum) with VY-AADC02 (3.6e+12 vg). The results showed tolerability in 15 patients, and there were no reported SAE after the therapeutic vector administration [137]. An interventional, Phase I study evaluated the safety and efficiency of VY-AADC01. Fifteen patients were enrolled, and IP administration was performed (NCT01973543). Interim results showed a well-tolerated treatment and the SAEs observed were independent of the therapeutic vector. The treatment showed an improvement of AADC activity [140].

A Phase I/II study (PROPEL; NCT04127578) evaluated safety, tolerability, immunogenicity, and biomarkers. Sixteen patients carrying a mutation in GB1 were enrolled and divided into two dose cohorts (Low dose or high dose) and sham. Patients were administered with LY3884961 via ICM injection.

GDNF protects against damage and strengthens brain cells producing dopamine. To evaluate the safety and efficacy of AAV2-carrying GDNF, a phase I clinical trial was initiated in 2019 and is still recruiting patients (NCT04167540). This study will dose a total of twelve patients assigned into two cohorts, an early stage, and a late stage, with six patients enrolled in each stage. The administration will be a single bilateral infusion into the putamen. The study is expected to be completed in 2027.

Decreased expression of GAD, a GABA-synthesizing enzyme, in the prefrontal cortex has been observed in PD. A phase I clinical trial testing the safety and efficacy of AAV2-GAD began in 2005 (NCT00195143). A total of twelve patients were enrolled. Patients received an infusion of AAV2-GAD into the subthalamic nucleus. This treatment was shown to be safe and tolerable and treated patients showed significant improvement in motor symptoms as measured by the Unified Parkinson’s Disease Rating Scale (UPDRS) scores. A phase II clinical trial to test the safety and efficacy of AAV-GAD began in 2008 (NCT00643890). The study enrolled 44 participants with advanced PD. Patients received AAV-GAD via bilateral infusion into the subthalamic nucleus at the dose of 1e+12 vg. Over the course of the six-month study, patients receiving AAV2-GAD had significant improvement in UPDRS scores compared to sham group scores [141]. A long-term follow-up study was initiated in 2011 (NCT01301573). However, both studies (NCT00643890 and NCT01301573) were terminated [141,142].

Neurturin (NRTN) is a neuroprotective neurotrophic growth factor. A group of three clinical trials using AAV2 carrying an NRTN transgene, called CERE-120, have been completed (NCT00252850, NCT00400634, NCT00985517). In these studies, a bilateral injection into the substantia nigra, putamen, and/or striatum was performed [143]. In 2006, the phase II study evaluating the safety and efficacy of CERE-120 administered via bilateral stereotactic injection into the putamen of patients with idiopathic PD began (NCT00400634). In total, 38 patients received a dose of 5.4e+11 vg, 4 of which did not complete the study, and seventeen patients received sham stereotactic surgery. Unfortunately, the study showed no significant difference between treated and sham groups [144]. Adverse effects were reported in thirteen out of thirty-eight total patients receiving CERE-120, three of whom developed carcinoma. Another CERE-120 clinical trial began in 2009 (NCT00985517). In this phase I/II trial, the safety and efficacy of CERE-120 in PD patients were evaluated using the following dose escalation: low dose (9.4e+11 vg), intermediary dose (2.4e+12 vg), and high dose (2.4e+12 vg) [145]. A total of fifty-seven patients were enrolled. Unfortunately, this study was also unsuccessful, as there were no significant improvements in symptoms of AAV treated compared to sham-treated patients [143].

### 4.4. Alzheimer’s Disease

Alzheimer’s disease (AD) is a neurodegenerative disease characterized by the progressive loss of memory and cognitive dysfunction. Around 6.2 million Americans aged 65 and older have been diagnosed with dementia. The number of individuals is projected to increase to 13.8 million by 2060 [146].

The classical hallmark of AD is the accumulation of amyloid beta (AB) plaques and neurofibrillary tau tangles. In most cases, the first areas of the brain to exhibit neurodegeneration as a result of the accumulation of AB and tau are the hippocampus and entorhinal cortex. Amyloid plaques, typically consisting of AB40 and AB42 peptides, accumulate in the extracellular space, and impact the synaptic processes, leading to neuronal dysfunction and degeneration. As AD progresses, other areas of the brain connected to the hippocampus and entorhinal cortex are impacted, such as the cerebral cortex, which has a detrimental effect on language, reasoning, and social behavior [147]. It is hypothesized that the severity of the disease is influenced by the level of AB accumulation, with more efficient degradation of plaques leading to a more moderate clinical phenotype in some patients [148].

Hyperphosphorylated tau (microtubule-associated tau protein-MAPT, tau) protein aggregation is identified in intracellular neurofibrillary tangles (NFTs) in neurons. In the healthy CNS, tau plays an important role in microtubule stabilization; however, in patients with AD, the accumulation of NFTs leads to impaired axonal transport and neuronal degeneration [149]. Of note, cytoplasmic inclusions of α-Syn localized in Lewy bodies within neurons have been identified in approximately 50% of sporadic AD patients and approximately 60% of familial AD patients.

One of the biological processes affected in AD is proteostasis, which is critical for the normal viability of cells. Several studies established that autophagy is the main pathway used in CNS proteolysis. Autophagy dysregulation and subsequent dysfunction of proteostasis in the brain result in the accumulation of misfolded protein and subsequent aggregation. The transcription factor EB (TFEB) regulates autophagy, proteostasis, and bioenergetics processes in the cells. TFEB is highly expressed in the CNS and plays a critical role in neuronal and astrocytic homeostasis [150]. In recent studies using several different transgenic AD mouse models, overexpression of TFEB diminished the proteotoxicity in the CNS and ameliorated cognitive and memory functions. Of note, a recent study has shown that TFEB overexpression enhanced skeletal muscle proteostasis. It is still unclear how the regulation of the TFEB pathway can interconnect the CNS and the skeletal muscle in AD. The understanding can help to discover a new target for translational medicine [150].

AD is a multifactorial disease, with the etiology of the disease still unknown. However, studies have shown that genetic mutations can impact the development of AD. Patients are classified into two main groups based on their age and the first appearance of cognitive symptoms. The first class (early onset) is identified in around 1–6% of patients around 60 to 65 years of age. The second group is identified as patients older than 65 years of age. A small group of patients have a familial history of the disease. Autosomal dominant inherited mutations in amyloid precursor protein (*APP*), presenilin 1 (*PSEN1*), or Presenilin 2 (*PSEN2*) genes have been implicated in the early development of AD and are even observed in 20-year-old patients [151]. Sporadic AD is typically observed in late-onset patients; however, additional copies of the *apolipoprotein E* 2 (*APOE2*) allele are associated with a higher risk of developing AD.

Because AD is a multifactorial disease, it is necessary to employ a multi-targeted approach to establish an optimal therapeutic approach. Several clinical trials have been conducted, but effective treatments are still lacking. Gene therapy approaches, including several AONs, mRNA, and microRNA have been designed to regulate the expression of specific genes implicated in AD [147]. There are several AON-based strategies for the treatment of AD. AONs an modulate the splicing of specific exons and restore normal protein expression levels using an exon-skipping strategy. AONs can be administered via systemic or CSF delivery. It is important to recognize the limitations of administration techniques when choosing the most efficient route of delivery. First, AONs must bypass the BBB to target cells in the brain after systemic delivery. A second challenge is protecting the small molecules from degradation by endogenous nucleases as they travel to their targets. To protect the AONs stability, direct chemical modification, conjugation with specific molecules, or encapsulation with non-viral or viral particles (such as AAV) can be utilized. To date, AONs have only been utilized in preclinical experiments.

The AONs in preclinical studies mainly target APP mRNA [152]. Alterations in APP have been identified in AD patients, causing cognitive dysfunctions. APP is cleaved by proteolysis, forming AB peptide, which has been identified as the main component of senile plaques in AD patients. Generated AB peptides can be between 38 to 42 amino acids in length, with AB40 and AB42 being the most prevalent. The elimination of AB peptide is a promising strategy for the treatment of AD. Various preclinical studies were performed in C57BL/6J mice, using AONs against APP mRNA. APP mRNA is a splice-switching antisense oligonucleotide (SSO), which leads to the skipping of the exon that encodes gamma-secretase cleavage sites in APP, leading to reduced generation of AB peptides. Notably, AON administration in C57BL/6J mice led to lower production of AB42 in the brain, including the hippocampus [153].

Another potential target for gene therapy is the tau protein, with more recent FDA approved clinical trials focusing on both AB peptides as well as tau protein [154,155]. A gene therapy trial from 2001 to 2012 utilized AAV2-carrying Nerve Growth Factor (NGF), a neurotrophic factor that induces growth and increases the survival of axons in peripheral sensory neurons (NCT00087789) [156]. NGF expression is correlated with the activation of functional markers in the brain [157,158]. Another clinical trial aimed at treating patients in early and mild stages with cognitive dysfunctions utilized AAV2-carrying Brain-Derived Neurotrophic Factor (BDNF) (NCT05040217). The delivery of BDNF in the CNS may provide a neuroprotective effect [159]. Another clinical trial is evaluating the effect of delivering APOE2 to a specific subset of AD patients using AAVrh10 via intrathecal CSF delivery (NCT03634007) (5e+10, 1.6e+11, 5e+11 gene copy (gc)/mL CSF). Currently, gene therapy via CNS delivery (ventricular, cisternal, or lumbar) has been studied as a promising treatment for AD [11]. Another recent Phase I study aims to assess the long-term safety of APOE4 homozygote AD patients (NCT05400330) who were IT administered (5e+10, 1.6e+11, 5e+11 gene copy (gc)/mL CSF) with rAAVrh.10-APOE2 (LX1001).

Telomerase (TERT) has a critical role in the maintenance of the telomeres. Shortened telomeres have been associated with AD. Preclinical trials utilizing AAV-delivered TERT showed an extension of telomeres, improvement in survival, and reversed signatures of aging [160]. A Phase I clinical trial of AAV-hTERT began in 2019 with the goal of extending the telomeres to prevent, delay, or reverse AD pathology. A total of five participants with AD were enrolled. Patients were injected with AAV-hTERT both IV and IT.

BDNF is a growth factor important in the memory circuits of the brain. A recent Phase I clinical trial evaluating the safety, tolerability, and efficacy of AAV2-BDNF for patients with AD or mild cognitive impairment (MCI) began in 2021 and is currently recruiting (NCT05040217). The estimated enrollment is twelve participants, six patients diagnosed with AD and 6 with mild cognitive impairment (MCI). Patients receive a stereotaxic injection of AAV2-BDNF into the brain parenchyma. Subjects will be followed during the two-year study and indefinitely after the conclusion of the study.

Significant advances have also been made in the treatment of NMDs for which specific examples are discussed in the following section.

### 4.5. Spinal Muscular Atrophy

Spinal Muscular Atrophy (SMA) is a neuromuscular disorder diagnosed mainly in children and adults. SMA follows autosomal recessive inheritance. The incidence of SMA is approximately 6000–10,000 live births leading to infant mortality [161,162]. SMA is characterized by the degeneration of spinal motor neurons, resulting in progressive muscle weakness in the body. In addition, paralysis, respiratory and cardiac failure, and premature death occured in children before two years of age in the most severe cases.

SMA is caused by homozygous (bi-allelic) deletions (around 95%), conversion, or loss of function mutations (around 5%) of the Survival Motor Neuron 1 (*SMN1*) gene. SMN forms part of a multiprotein complex with important functions in both spliceosome snRNP assembly and for the pre-mRNA splicing process [163]. The SMN protein is ubiquitously expressed, and its absence is embryonic lethal. SMA patients carrying mutations in SMN1 survive only thanks to the production of a small amount of full-length protein by the SMN2 gene. Unfortunately, SMN2 carries a silent mutation in exon 7, which impairs splicing and leads to the predominant production of a truncated and unstable protein isoform and fewer amounts of full-length protein. Motor neurons in the spinal cord are particularly vulnerable in SMA and previous publications have shown that this is in part due to the very low production of full-length protein from SMN2 in this particular cell type [164].

Patients can carry variable numbers of SMN2 copies, which modify the SMA phenotype as they allow gradually increasing amounts of SMN protein to be produced. According to disease phenotype and progression, SMA has been categorized into different types, with type 0 being the most severe, with the onset of symptoms in utero to type 4, which become affected only in early adulthood. Although no perfect genotype–phenotype correlation exists, generally patients with one or two copies of SMN2 will display SMA type 0 or 1, while patients with four or more SMN2 gene copies will display type 4 or might even be asymptomatic.

In 2016, the first effective treatment for SMA, an SMN2-directed AON that allows to increase exon 7 inclusion and therefore SMN protein production by the SMN2 gene was FDA approved for use in children and adults (Spinraza™, Manufactured by Biogen, Inc., Cambridge, MA, USA). Because Spinraza is not able to cross the BBB, a lumbar intrathecal CSF delivery was chosen for the route of administration. The initial loading dose of the drug is administered over a period of four visits and then administered three times a year moving forward. Repeated dosing is necessary as AONs degrade over time. Treatment with Spinraza led to increased expression of full-length SMN protein in patients and improved motor function. Spinraza was the first truly disease-modifying treatment for SMA that received FDA approval.

Since Spinraza, two additional disease-modifying treatments have been approved, Risdiplan and Zolgensma. Risdiplam Evrysdi^®^, (Roche), is the first oral drug for this disease. SMA type 1, type 2, and type 3 patients are eligible for treatment from 2 months of age to 60 years. The mechanism of this drug is similar to the AON Spinraza in that it allows for the inclusion of exon 7 in the *SMN2* mRNA, thus increasing the SMN protein level in the patients leading to improvements in survival, motor skill, and pulmonary functions [165].

Onasemnogene abeparvovec, Zolgensma™ (Novartis) is the second FDA-approved AAV gene therapy. This is an AAV9-mediated gene replacement strategy that restores functional SMN expression via the delivery of the *SMN* coding sequence. Expression is mediated by the CMV-enhancer chicken beta actin promoter. Zolgensma is administered via IV delivery at a dose of 1.1e+14 vg/kg and is approved in the US for the treatment of children up to 2 years of age.

The initial clinical trial (START; NCT02122952) included 14 pediatric patients, less than 2 years of age, with bi-allelic mutations in the *SMN1* gene. The treatment was overall well-tolerated, and several patients had remarkable motor outcomes, including the ability to stand and walk unassisted, while untreated patients would be completely paralyzed, ventilator dependent, or diseased at that age. Survival has also been extended well beyond the two-year life expectancy [166]. Following the initial START trial, a Phase III multicenter safety and efficacy trials (STR1VE; NCT03306277 and NCT03461289) were conducted in the US and the EU. The success of these trials ultimately led to the FDA and EMU approval of the treatment. Subsequently, an additional clinical trial (SPR1NT; NCT03505099) was initiated that evaluated treatment efficacy in presymptomatic patients that have two or three copies of *SMN2* [167]. Interim data from the SPR1NT study show the achievement of age-appropriate milestones in patients treated presymptomatically with Zolgensma [168].

As mentioned above, due to safety concerns with high IV dosing, Zolgensma is only available for patients under 2 years of age in the US. However, additional clinical trials were conducted to evaluate CSF delivery for the treatment of older SMA patients (STRONG; NCT03381729). This Phase I/II trial enrolled patients with bi-allelic deletion of SMN1 and three copies of *SMN2* who are able to sit unassisted prior to treatment. The primary outcome was to evaluate efficacy, safety, and tolerability [169]. This trial was placed on a temporary, partial hold in 2019 due to concerns about DRG pathology in non-human primates after a study was published that reported this evident pathology post-lumbar AAV delivery in NHPs. No indication of DRG pathology was seen in the clinical trial, according to statements released by Novartis and 50% of the patients showed a remarkable increase in strength within 2 months post-treatment. The hold was lifted in 2021 and Novartis is currently planning a global Phase III Pivotal registration-enabling study [170].

### 4.6. Duchenne Muscular Dystrophy

Duchenne muscular dystrophy (DMD; OMIM: 310200) is a rare disorder occurring mainly in boys, which has an incidence of 1 in 5000 live male births [171,172]. DMD is an X-linked recessive genetic disorder caused by mutations in the *DMD* gene resulting in a lack of expression of dystrophin. Around 30% of the mutations are nonsense, missense, and frameshift, among others. Deletions of single or multiple exons are identified in 60% of cases. Duplication of one or multiple exons is around 6–11% of DMD patients. These mutations can result in an out-of-frame transcript and coding of a truncated and nonfunctional protein leading to a more severe phenotype called DMD, while the in-frame transcript results in an expression of shorter forms of partially functional dystrophin-associated with a milder Becker muscular dystrophy ((BMD); OMIM: 300376) phenotype. There is also an intermediary phenotype called intermediary muscular dystrophy, which shares some characteristics of both types of phenotypes mentioned above [173,174,175].

The symptoms observed in DMD patients between the first months and one year of age are motor deficits and weakness in the muscles. Other common symptoms observed in patients between 3 to 5 years of age are difficulty walking or climbing stairs. In addition, some patients present cognitive deficiency. Patients between 9 to 14 years of age lose ambulation and generally become wheelchair-bound. During the progression of the disease, the patients show failure in the respiratory and cardiac system from teens to 20 years of age. Ventilation support increases the survival of these patients. Patients die in the 2nd or 3rd decades of their life due to respiratory and cardiac failure. The cardiac system is compromised due to sub endocardial fibrosis and its impact on the dilation of the cavities. Ventricular-assisted devices are necessary for these patients. BMD patients survive up to 40 years of age [176].

Different methods are used to diagnose patients with DMD. To evaluate the damage in the muscle fibers, the most common evaluation is a biochemical analysis to measure the level of serum Creatine Kinase (CK); high levels are correlated with necrosis of the fibers. Levels of other proteins such as LDH, fatty acids, carnosine taurine and creatine, latent TGF beta-binding protein 4—LTBP4 genotypes are also used in the diagnosis and monitoring of the progression of the diseases. Serum levels of matrix metalloproteinase 9 (MMP-9), myostatin (GDF-8), and follistatin are non-invasive biomarkers used in the diagnosis of DMD [177].

Dystrophin contains 79 exons, a large size of 14 kb (mRNA), and is an essential protein in the sarcolemma Dystrophin-Associated glycoprotein Complex (DAG). DAG has a critical role in the motor function of muscle fibers. The absence of dystrophin in the DAG results in membrane instability and disruption of calcium homeostasis, increasing the intracellular concentration of calcium and activating the calcium-dependent proteases. Together, these events lead to the failure of the contraction process of these fibers and posterior necrosis [178].

The damage in these fibers can activate a cycle of degeneration/regeneration by activating the satellite cells, which are adult muscle Stem Cells (mSCs). mSCs are able to differentiate into myocytes and initiate the regeneration and substitution of non-functional fibers for new fibers. Infiltration of immune cells, fibrosis, and necrosis are identified in both skeletal and cardiac muscles. Tissues with high cycles of degeneration/regeneration increase the number of Central Nucleation (CN) in the fibers. The quantification of CN is used in several studies to correlate with the degree of fiber damage. In the progression of the disease, the satellite cells can enter into an exhaustion state, losing the capacity for regeneration. Because mSCs cannot regenerate the fibers, it is common to observe fat cells replacing fibers in patients with DMD [179]. There are multiple mouse models of DMD, carrying different types of mutations, such as mdx mice and dup2 as well as a canine model called Golden Retriever Muscular Dystrophy (GRMD) [180].

One out of three patients with DMD presents with cognitive development problems; in some cases, the IQ is lower than in healthy boys. Several studies have focused on the role of dystrophin in brain function. Different dystrophin isoforms are expressed in the brain, but to date, the role of these protein isoforms remains to be elucidated.

The lack of dystrophin expression due to a mutation in different isoforms such as Dp140, 71, and 427 are implicated in cognitive dysfunction and neurodevelopmental disorders, including autism, attention deficit hyperactivity disorders, and DMD, among others. Nevertheless, how these isoforms affect and govern symptoms in these different diseases remains unknown.

Multiple clinical trials for DMD treatments are either completed or ongoing, including treatments that induce exon skipping in different exons of the *DMD* gene. U7 small nuclear RNA (snRNA) is used to induce the skipping of Exon 2 (NCT04240314), Casimersen (SRP-4045) induces the skipping of Exon45 (NCT04433234, NCT04179409, NCT03532542), Eteplirsen (AVI-4658) (NCT03218995, NCT04179409, NCT03992430, NCT03985878), Drisapersen (NCT02636686), Vesleteplirsen (SRP-5051) (NCT03675126, NCT04004065), and Suvodirsen (NCT03907072) are used to induce skipping of Exon 51. SRP-5051-2 is a new drug generation to skip exon 51 more efficiently (NCT04004065). A study recently started utilizing DYNE-251 (NCT05524883) to test the safety profile and showed efficient exon skipping in non-human primates, mainly in the heart, diaphragm, and muscles.

Furthermore, other treatments induce exon skipping in Exon53 utilized Golodirsen (SRP-4053) (NCT04179409, NCT03532542). A study with Golodirsen (NCT02310906) reported evidence of long-term efficiency and safety of the treatment compared with the control cohort [181]. Another treatment is using Viltolarsen (NCT03167255, NCT04060199) [182]. An additional study (ESSENCE), Phase III (NCT02500381), showed that Casimersen was well-tolerated with mild and unrelated SAE in the patients [124,183]. Recently a study in Phase II was completed to treat DMD patients carrying duplication of exon 45, exon 51, or exon 53. Patients were treated with Casimersen, Eteplirsen, or Golodirsen, respectively (NCT04179409). This study was a 48-week open-label study to evaluate the safety of these treatments. A study, Phase III aimed to evaluate the efficacy and safety of Ataluren in DMD patients with nonsense mutation (NCT01826487). As indicated by the multitude of ongoing and completed clinical trials, AAV-mediated gene therapy approaches are promising alternative treatments [182].

Micro- or minidystrophins are truncated dystrophin protein sequences that have been designed to carry out the most essential functions of the protein but are much smaller and therefore fit in AAV viral vectors for delivery. Three clinical trials using different versions of microdystrophin demonstrated efficiency to restore the function of muscle fibers are ongoing by Sarepta Therapeutics, Pfizer, and Solid Biosciences; however, some differences were identified between the microdystrophins and the full-length dystrophin and AAV serotypes.

A Phase I/II study administered the AAV9-CK8.micro-dystrophin (SGT-001) via systemic injection in a low dose (5e+13 vg/kg) cohort of three patients SAE were recorded in a patient; however, it was resolved. Patients showed restoration of the normal dystrophin in muscles. The second cohort of three patients received a high dose (2e+14 vg/kg). A patient showed SAE, which was resolved by the administration of oral glucocorticoids (IGNITE-DMD, NCT03368742) [184]. Another Phase I study administered systemically AAV9-micro-dystrophin (PF-06939926) low dose (1e+14 vg/kg) in a cohort 1B and a high dose (3e+14 vg/kg) in a second cohort. Two patients presented with SAE. While these two SAEs were severe, all were fully resolved within 2 weeks. Those indicates that close monitoring and early intervention can help mitigate the effects of complement activation response against AAV. DMD patients showed normal dystrophin expression in muscles in the high-dose cohort (NCT03362502) 222. A Phase I/II study is evaluating the safety of AAVrh74.MHCK7. micro-dystrophin (Delandistrogene moxeparvovec, SRP-9001). A cohort of four DMD patients was injected via IV (2e+14 vg/kg). Three patients presented an elevated level of enzyme gamma-glutamyl transpeptidase in the liver, which was solved with steroid treatment (NCT03375164). A second study, Phase II, complemented the last clinical trial by adding the placebo group (NCT03769116). The findings showed that patients presented with SAE, which was solved [185,186].

### 4.7. Pompe Disease

Glycogen storage disease type 2 (OMIM # 232300), also known as acid maltase deficiency or Pompe disease, is a rare, inherited, fatal NMD. The incidence is around 1 in every 40,000 live births and the prevalence is 1 in 8700 patients. Pompe disease is a metabolic disorder affecting patients of different ages, characterized by progressive, smooth, cardiac, and skeletal muscle damage and weakness. In recent years, a CNS component of Pompe disease has been identified, as patients can present with behavioral, sensory, and cognitive dysfunction. Autopsy results also show brain and spinal cord abnormalities in patients with Pompe disease [187,188].

Pompe disease is an inherited autosomal recessive disease. Mutations in the *GAA* (acid alpha-glucosidase) gene have been implicated in the development of disease, with most mutations resulting in an activity deficit of acid alpha-glucosidase enzyme (acid maltase). The partial or complete absence of the acid alpha-glucosidase enzyme activity leads to a reduction in the breakdown of the glycogen stored in the lysosomes, resulting in the accumulation of glycogen inside lysosomes. The accumulation of glycogen can increase the number of lysosomal compartments and affects the viability of these cells [189,190]. The accumulation of glycogen due to a deficiency of alpha-glucosidase enzyme is observed in many tissues, but a deficiency in muscle fibers can lead to dysfunction of cardiac and skeletal muscles.

Pompe disease patients are classified into subgroups based on their age, rate of progression, and first symptoms, including cardiac deficiency. Taken together, these parameters can determine the severity of the disease. The level of deficiency of alpha-glucosidase enzyme activity positively correlates with the severity of the disease. Thus, patients who have a complete absence of alpha-glucosidase enzyme activity, and present symptoms before 6 months and up to one year of age are the most severely affected and are classified as having early Infantile-Onset Pompe Disease (IOPD). Patients can present with disease symptoms as early as one month of age [191]. IOPD patients often present with severe hypotonia, cardiomegaly, feeding issues, progressive muscle weakness, and loss of motor function. In addition, patients also present with hepatomegaly and macroglossia. The consequence of the cardiac dysfunction of untreated IOPD patients causes life-threatening complications by the age of 12 to 18 months and premature death.

Another subgroup of patients is non-classic infants. These patients are also diagnosed before one year of age, but most do not exhibit cardiac failure. The symptoms of non-classic infants typically manifest a few months later than infantile-onset patients. These symptoms include difficulty walking, crawling, and sitting. These patients also exhibit progressive muscle weakness and life-threatening breathing issues in early childhood [192].

Late-onset or juvenile/adult patients are diagnosed after one year of age. These patients show a partial function of the alpha-glucosidase enzyme and thus have a less severe phenotype. The symptoms are identified around 12 years of age or later, and patients typically do not have symptoms associated with myocardiopathy. Progressive weakness in the legs and the trunk begins in late childhood, adolescence, or adulthood. As the disease progresses, these patients present with respiratory system dysfunction [193]. Because symptoms vary widely across patients, there is a database called Human Phenotype Ontology (HPO) which contains more information about the symptoms.

Diagnosis is difficult due to the lack of efficient biomarkers; however, there are two methods that can be used to diagnose these patients. First, mutations can be identified and confirmed by sequencing. Second, the activity of the enzyme can be measured in the plasma (blood) or in different tissues such as skin fibroblasts and muscles.

In 2006, the first ERT for patients with infantile onset was approved by the FDA. Recombinant human alglucosidase alfa (Myozyme^®^ and Lumizyme^®,^ which are manufactured by Genzyme Corporation), which is manufactured by Sanofi Genzyme. Though these treatments are beneficial, they have limitations. ERT reduces glycogen accumulation in the lysosomes. The drug is administered via biweekly IV infusion in patients between 41 to 60 years of age. ERT treatment was able to restore muscle function, improve walking distance, and improve cardiac and respiratory function, thereby increasing life expectancy [194]. A limitation of ERT is the patient’s immune response [195].

Another limitation is that ERT does not efficiently target the skeletal muscle—muscle fibers possess a low quantity of a specific receptor able to internalize the enzyme [196]. Finally, a third limitation is that ERT cannot treat the CNS symptoms of these patients, as alpha-glucosidase cannot bypass the BBB [197].

A new ERT called neo-GAA avalglucosidase alfa (neo-GAA) (NeoGAA^®^), also manufactured by Sanofi Genzyme, is currently being evaluated. A clinical trial evaluated safety and tolerability in patients less than 18 years of age (NCT02782741). The study demonstrated efficacy after independent ambulation. Survival was also improved, and the treatment was well-tolerated [198].

An ongoing Phase I/II clinical trial is testing a recombinant human alpha-glucosidase (ATB200) with a high content of mannose-6-phosphate (M6P), which allows binding with high affinity with surface receptors in myoblast and increased uptake in skeletal muscles (NCT02675465). ATB200 is co-administered with N-deoxynojirimicine (NB-DNJ). NB-DNJ is a pharmacological chaperone (PC) AT2221, which can improve the pharmacological features, acting as a “protective” molecule of ATB200 after administration. The pre-clinical studies were performed in nonhuman primates (NHP), rat, and mouse via IV administration. The combination therapy showed higher efficiency in reducing the glycogen compared with a single administration of alpha-glucosidase [198,199].

Currently, ERT is the only therapy approved by the FDA for patients with Pompe disease. Due to the short half-life of ERT, activation of the patient’s immune system against the foreign protein negatively influences the efficiency of the treatment. As the immune response induces tolerance to the treatment, several reports have suggested an alternative treatment based on utilizing AAV-mediated therapy. However, higher doses of AAV have also been shown to induce tolerance.

A preclinical comparison study evaluated the efficiency of treatment using AAV9-CAG-*hGAA* or AAVrh10-CAG-*hGAA* via CSF delivery. A single ICM injection of either AAV9-CAG-*hGAA* or rAAVrh10-CAG-*hGAA* was performed in Gaa-deficient mice and the efficiency of the treatment four months post-injection was evaluated. The results showed reduced levels of glycogen in the CNS using both rAAVs, with the exception of motor neurons in the cervical and lumbar spinal cord. In these regions, AAV9-CAG-hGAA-treated mice presented higher levels of glycogen accumulation compared with AAVrh10-CAG-hGAA-treated animals [200].

There are currently five clinical trials listed on clinicaltrials.gov, all evaluating AAV-mediated gene replacement therapy. A clinical trial utilizing AAV2/8 to deliver functional GAA to hepatocytes began in 2018 and is currently recruiting patients (NCT03533673). In this Phase I/II trial, an estimated enrollment of eight patients will receive the gene therapy via IV administration at three different dose levels. Results were presented from three patients treated with a low dose of ACT-101, showing an increase in GAA expression in the muscles after 1 year of treatment. ACT-101 is safe and well-tolerated. However, no significant improvement was identified after evaluating walking ability and lung function. SAEs were observed in one patient who was resolved with treatment [201]. A second Phase I/II study using rAAV1 to deliver a normal copy of GAA to cells of the diaphragm began in 2009 (NCT00976352). Nine patients received an IM injection into the diaphragm (n = 3, 1e+12 vg or n = 6, 5e+12 vg). Patients developed antibodies against AAV1 and the GAA transgene and unfortunately, there were no conclusions concerning the therapeutic efficacy of this treatment. A Phase I/II clinical trial evaluated the toxicology, biodistribution, and efficacy of a re-administration of rAAV9 (4.6e+13 vg per TA muscles) carrying codon-optimized GAA (coGAA) to the TA (NCT02240407). In this study, two patients were enrolled and received the therapy or sham control via IM injection of the TA of one leg. Four months later, patients were re-dosed with the therapy or sham injection via IM injection of the TA of the contralateral leg. Patients also received immunosuppressive therapy consisting of Sirolimus (Rapamycin), Rituximab (Rituxan^®^), and Diphenhydramine (Benadryl^®^) to ablate B-cells before each injection. To evaluate safety, tolerability, and efficacy of AAV-delivered GAA (SPK-3006) in adults with moderate, late-onset Pompe disease receiving ERT, a phase I/II clinical trial is currently recruiting (RESOLUTE—NCT04093349). An estimated enrollment of thirty patients will receive an IV injection of the therapy in dose-level cohorts. The FORTIS phase I/II clinical trial is still recruiting (NCT04174105). In this study, an estimated enrollment of twelve patients across three dose cohorts will receive AAV-delivered GAA (AT845, AAV8) via IV infusion (low dose: 3e+13 vg/kg, intermediate dose: 6e+13 vg/kg, high dose: 1e+14 vg/kg).

## 5. Future Perspectives

The study of CSF delivery is critical to understanding the limitations and advantages of this route of delivery for the treatment of NDD and NMDs. However, there are diseases that urgently need a new alternative approach based on their complexity. The efficiency of the treatment and biodistribution studies of CSF delivery compared with systemic IV delivery have been investigated in mouse models, large animals, and humans in CNS disorders and NMDs. The understanding of the genetic background associated with the disease and the experimental protocols established to treat mouse models of NDD and NMD, as well as the use of large animals, have been extremely helpful for the translational of novel therapeutics for these types of rare diseases. Clinical trials in phases I/II or III, as well as FDA-approved novel treatment options, have shown promising results for patients with NDDs and NMDs. The combination of AAV-mediated gene therapies, as well as AONs and administration routes, still need to be better studied in humans. Together, combined efforts in AAV capsid engineering, promoter and enhancer design, as well as exploring new delivery routes and systems, will help improve the safety as well as the efficacy of novel treatments for debilitating NDDs and NMDs. 

## Figures and Tables

**Table 1:** List of ongoing gene therapy-based clinical trials for NDDs and NMDs. Intraparenchymal (IP), intrathecal (IT), intra-cisterna magna (ICM), intracerebroventricular (ICV), intravitreal (IVT), intravenous (IV), (NHGRI) National Human Genome Research Institute.

Gene Therapy	Delivery Route	Sponsor	Phase	Identifier	Participants Estimated Enrollment	Duration
GM1						
AAV9–GLB1	IV	NHGRI	Phase I/II	NCT03952637	45	2019–2027
AAVhu68-GLB1	ICM	Passage Bio, Inc.	Phase I/II	NCT04713475	20	2021–2029
AAVrh.10–GLB1	ICM	Lysogene	PhaseI/II	NCT04273269	16	2021–2030
Batten disease						
AAV9-CLN3	IT	Amicus Therapeutics	Phase I/II	NCT03770572	7	2018–2023
AAV9–CLN5	ICV, IVT	Neurogene Inc	Phase I/II	NCT05228145	3	2022–2028
AAV9–CLN7	IT	Benjamin Greenberg	Phase I	NCT04737460	4	2021–2029
PD						
AAV2–AADC	IP (striatum)	Neurocrine Biosciences	Phase II	NCT03562494	85	2018–2023
AAV2–GDNF	IP (putamen)	Brain Neurotherapy	Phase I	NCT04167540	12	2020–2027
AAV9–GCase	ICM	Prevail Therapeutics	Phase I/IIa	NCT04127578	24	2020–2028
AD						
AONS–MAPT	IT	Ionis Pharmaceuticals, Inc.	Phase I/II	NCT03186989	44	2017–2022
AAV2-BDNF	stereotactic administration-brain	Mark Tuszynski	Phase I	NCT05040217	12	2021–2025
AAVrh.10-APOE2	IT	Lexeo Therapeutics	Phase I	NCT03634007	15	2019–2024
AAVrh.10-APOE2	IT	Lexeo Therapeutics	Phase I	NCT05400330	15	2022–2027
SMA						
AONS–SMN2	IT	Biogen	Phase II	NCT02386553	25	2015–2025
AONS–SMN2	IT	Biogen	Phase III	NCT02594124	292	2015–2023
AONS–SMN2	IT	NYU Langone Health	Early Phase I	NCT04050852	0	2019–2022
AONS–SMN2	IT	Biogen	Phase II/III	NCT04089566	145	2020–2023
AONS–SMN2	IT	Biogen	Phase III	NCT04729907	172	2021–2026
DMD						
AAV9–DMD	IV	Pfizer	Phase III	NCT04281485	99	2020–2029
AAVrh74.MHCK7. micro-dystrophin	IV	Serapta Therapeuitics, Inc.	Phase III	NCT05096221	120	2021–2024
AAV9-CK8.micro-dystrophin	IV	Solid Biosciencese, LLC	Phase I/II	NCT03368742	16	2017–2028
AAVrh74.MHCK7. micro-dystrophin	IV	Serapta Therapeuitics, Inc.	Phase I/II	NCT03375164	4	2018–2023
AAVrh74.MHCK7. micro-dystrophin	IV	Serapta Therapeuitics, Inc.	Phase II	NCT03769116	41	2018–2026
AAV9-micro-dystrophin	IV	Pfizer	Phase I	NCT03362502	23	2018–2026
scAAV9.U7.ACCA	IV	Megan Waldrop	Phase I/II	NCT04240314	3	2020–2025
Pompe Disease						
AAV2/8–GAA	IV	Asklepois Biopharmaceutical	Phase I/II	NCT03533673	13	2018–2028
AAV–GAA	IV	Spark Therapeutics	Phase I/II	NCT04093349	30	2020–2027
AAV8–GAA	IV	Audentes Therapeutics	Phase I/II	NCT04174105	12	2020–2027

**Table 2:** AAV serotypes, receptors and co-receptors, expression efficacy, and targeted organs. Abbreviations: HSPG: heparan sulfate proteoglycan, FGFR-1: fibroblast growth factor receptor 1, HGFR: hepatic growth factor receptor, PDGFR: platelet-derived growth factor receptor, LamR: 37/67 KD laminin receptor, EGFR: epidermal growth factor receptor. SM: Skeletal muscle, CM: cardiac muscle, HCC: hepatocellular carcinoma. AAVR: Adeno-associated virus (AAV) receptor. PKD: Glycosylated protein containing five polycystic kidney disease (PKD) with repeat domains in its extracellular domain.

Serotype	Host	Receptor	Co-Receptor	AAVR (Recognition PKD Receptors)	Tropism	Organ Tropism	Type of Transport
AAV1	Human/ Monkey	2,3N/2,6N-sialic Acid	Unknown	PKD1/2 or PKD2 [40]	medium	SM, CNS, liver, retina, pancreas, heart, airways, CM	retrograde anterograde
AAV2	Human	HSPG	FGFR-1, integrin, HGFR, LamR	PKD2	low	SM, CNS, liver, kidney, retina	retrograde anterograde
AAV3	Human	HSPG	FGFR-1, HGFR, LamR,	PKD, AAV3B [41]	low	SM, HCC, liver	N/A
AAV4	Monkey	2,3O-sialic acid	Unknown	N/A	low	CNS, retina, lung, kidney	N/A
AAV5	Human	2,3N-sialic acid	PDGFR	PKD1 [40]	medium	SM, CNS, airway, retina	retrograde anterograde
AAV6	AAV1/ AAV2 hybrid	2,3N/2,6N-sialicAcid, HSPG	EGFR	PKD	medium	SM, heart, airway	retrograde
AAV7	Monkey	N-sialic acid	PDGF	N/A	high	SM, retina, CNS, liver	
AAV8	Monkey	Unknown	LamR	PKD1/2 or PKD2	high	SM, CNS, liver, retina, pancreas, heart, and kidney	retrograde anterograde
AAV9	Human	N-galactose	LamR	PKD2 [42]	high	SM, CNS; liver, heart, lung, pancreas, retina, testes, kidney	retrograde anterograde
AAVrh.10	Monkey	sulfated N-acetyl-lactosamine	Unknown	N/A	high	SM, CNS; liver, heart, lung, pancreas, retina, kidney	retrograde anterograde
AAV10	Monkey	Unknown	Unknown	N/A	high	SM, CNS; liver	N/A
AAV11	Monkey	Unknown	Unknown	N/A	medium	spleen	N/A
AAV12	Monkey	Unknown	Unknown	N/A	medium	Submandibular glands, liver	N/A
